# Protein–DNA binding sites prediction based on pre-trained protein language model and contrastive learning

**DOI:** 10.1093/bib/bbad488

**Published:** 2024-01-03

**Authors:** Yufan Liu, Boxue Tian

**Affiliations:** MOE Key Laboratory of Bioinformatics, State Key Laboratory of Molecular Oncology, School of Pharmaceutical Sciences, Tsinghua University, Beijing, 100084, China; MOE Key Laboratory of Bioinformatics, State Key Laboratory of Molecular Oncology, School of Pharmaceutical Sciences, Tsinghua University, Beijing, 100084, China

**Keywords:** protein–DNA interaction, pre-training, contrastive learning

## Abstract

Protein–DNA interaction is critical for life activities such as replication, transcription and splicing. Identifying protein–DNA binding residues is essential for modeling their interaction and downstream studies. However, developing accurate and efficient computational methods for this task remains challenging. Improvements in this area have the potential to drive novel applications in biotechnology and drug design. In this study, we propose a novel approach called Contrastive Learning And Pre-trained Encoder (CLAPE), which combines a pre-trained protein language model and the contrastive learning method to predict DNA binding residues. We trained the CLAPE-DB model on the protein–DNA binding sites dataset and evaluated the model performance and generalization ability through various experiments. The results showed that the area under ROC curve values of the CLAPE-DB model on the two benchmark datasets reached 0.871 and 0.881, respectively, indicating superior performance compared to other existing models. CLAPE-DB showed better generalization ability and was specific to DNA-binding sites. In addition, we trained CLAPE on different protein–ligand binding sites datasets, demonstrating that CLAPE is a general framework for binding sites prediction. To facilitate the scientific community, the benchmark datasets and codes are freely available at https://github.com/YAndrewL/clape.

## INTRODUCTION

The interaction of protein and ligands dominates almost all the life activities in organisms, including interactions of protein–protein, protein–small molecules and protein–nucleic acids. As carriers of genetic information, DNA molecules binding with proteins play a crucial role in many biological processes, including DNA transcription, replication, expression, signal transduction and metabolism [1, 2]. In prokaryote and eukaryote species, approximately 3% and 7% of genomes encode DNA-binding proteins, respectively [3]. Transcription factors (TFs) are a representative group of DNA-binding proteins that regulate transcription by binding to specific DNA sequences known as motifs. TFs are involved in various biological processes, including immune response [4] and maintenance of pluripotency of stem cells [5], and the dysfunctions of TFs are related to numerous human diseases, such as various types of cancer and neurodegenerative diseases [6, 7]. In addition, other DNA-binding proteins such as histone, DNA polymerase and DNA topoisomerase also play critical roles in biological activities and are associated with human diseases [8, 9].

Identifying the DNA-binding sites of a protein is the initial step for modeling protein–DNA binding properties. Several experimental approaches have been developed for identifying protein–DNA interaction *in vivo* or *in vitro*, such as systematic evolution of ligands by exponential enrichment and chromatin immunoprecipitation [10, 11]. In addition, structural biology approaches have been applied to determine the DNA-binding residues and areas, including X-ray crystallography and nuclear magnetic resonance. Although experimental methods based on molecular biology have made significant contributions over the past few decades, these methods are time consuming and resource intensive. Therefore, computationally predicting DNA-binding residues with machine learning methods is attractive.

The vital step in building a predictor is representation learning, where discriminative features play a crucial role in improving model performance. Typically, models utilize features extracted from a collection of protein sequences to fully leverage evolutionary information. The commonly used methods involve PSI-BLAST [12] and HHblits [13], which produce multiple sequence alignment described as a position-specific scoring matrix (PSSM). Extensive studies show that evolutionary information leads to significant improvement in DNA-binding prediction tasks [14, 15]. The secondary structure information of the given protein can also be applied as the initial feature, which can be generated by DSSP [16] using protein structure or PSIPRED [17] using protein sequence. Several models have been developed to complete the task and can be roughly divided into sequence-based and structure-based models. Sequence-based models extract features from protein sequences alone, while structure-based models use features of crystal protein structures. BindN [18] used several amino acid properties as sequence features, applying a support vector machine (SVM) model to classify the DNA-binding residues; BindN+ [14] improved the model performance by adding the PSSM feature. Currently, advanced predictors are focused on deep learning methods, with DeepDISE [19] and DBPred [15] using a convolutional neural network (CNN) as the classifier, EL_LSTM [20] applying a recurrent neural network (RNN) as the backbone network and ProNA2020 [21] using a multi-layer perceptron (MLP). A few models start with predicted protein structures or experimentally solved structures. NucBind [22] predicted protein structures by template-based models and then used an SVM-based machine learning method to complete the downstream prediction. GraphBind [23] integrated sequence-based and structure-based features, employing graph neural network (GNN) as the classifier.

Protein structures contain all the necessary information derived from the protein sequence. Hence, in general, structure-based models demonstrate better performance than sequence-based models_ENREF_31. However, to ensure model performance, structure-based models require accurate protein structures as input [23, 24]. Consequently, the prediction of DNA-binding sites based on protein sequences remains an important and pressing research problem. Currently, the performance of existing sequence-based models is still unsatisfactory for practical application, and the feature extraction process often relies on manual design, which fails to generate a refined initial representation [24]. As a result, there is a pressing need to develop an end-to-end model without using handcrafted features. Pre-training and contrastive learning are two widely used representation learning techniques. Pre-training utilizes the information of a large scale of unlabeled data to train the model in an unsupervised manner and transfers the model parameters to downstream tasks for fine-tuning or feature extraction [25], which is widely used in tasks such as protein property and structure prediction [26, 27]. Contrastive learning aims to discover a representation space where samples from the same class are close to each other, while those from the different classes are distant, which effectively enhances the representative ability of protein embeddings and the model performance in related fields such as protein–ligand interaction prediction [28, 29]. In addition, the vast majority of models did not take into account data imbalance issues, which we complemented by applying class-balanced focal loss in our task.

In this study, we integrated pre-training and contrastive learning techniques to devise the Contrastive Learning And Pre-trained Encoder (CLAPE), which enabled the prediction of ligand-binding sites of a protein sequence. CLAPE received the raw protein sequence data in FASTA format and generated ligand-binding sites without pre-computing manually designed features, which is an end-to-end prediction model. Specifically, we trained CLAPE-DB on DNA-binding datasets and demonstrated that it surpassed current sequence-based models by learning a discriminative embedding space. In addition, we illustrated that CLAPE could serve as a general framework for predicting ligand-binding sites exclusively based on protein sequence information, thereby improving the comprehension of the feature extraction process and the development of the model architecture for future research.

## RESULTS

### The model architecture of CLAPE

The existing models for identifying protein–DNA binding sites could be divided into two categories. The first category combines handcrafted features and classification models (Supplementary Figure 1 available online at http://bib.oxfordjournals.org/). Handcrafted features may include amino acid physicochemical properties and protein structural information, while the models may include machine learning models such as support vector machine and random forest. The second category aims to predict DNA-binding sites in an end-to-end fashion (Supplementary Figure 1 available online at http://bib.oxfordjournals.org/) and often employs large-scale deep learning models. However, the first approach typically necessitates laborious manual feature extraction processes, while the second approach demands high computational resources and training time.

**Figure 1:**
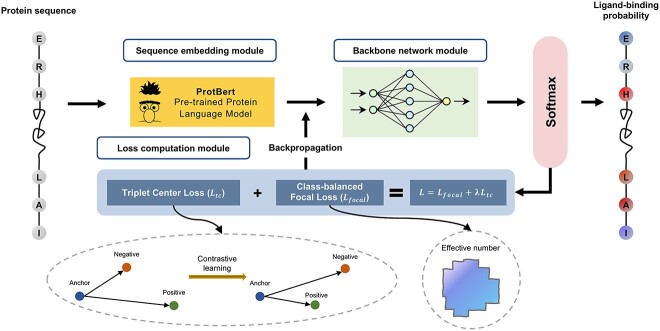
Schematic representations of the overall architecture of the CLAPE model. The CLAPE model consists of three primary modules: the sequence embedding module for generating protein sequence representations using a pre-trained protein language model named ProtBert; the backbone network module for downstream processing of protein embeddings, which is flexible by applying different types of mainstream neural networks such as MLP, CNN, RNN and GNN; and the loss computation module for computing binary classification class-balanced focal loss and contrastive loss for backpropagation to update the model parameters of the backbone model. In the triplet center loss part, for an anchor sample, the positive sample refers to the learnable cluster center within the same class as the anchor sample, while the negative sample corresponds to that from a different class as the anchor sample. In the class-balanced focal loss part, the effective number is a coefficient that weights different sample numbers. All strategies share a classification module for generating classification scores between 0 and 1, which contains a Softmax function.

We took advantage of both approaches to propose CLAPE, a protein–ligand binding sites prediction framework to generate the binding probabilities of a given protein sequence. The overall architecture of CLAPE is depicted in Figure 1, which comprised three main modules: the sequence embedding module, the backbone network module and the loss computation module. The sequence embedding module utilized ProtBert [30], a pre-trained protein language model, to encode protein sequences and generate features. The features were then passed through the backbone network, which in CLAPE was a four-layer 1DCNN. The loss computation module employed a contrastive loss function guided by binary classification loss. Finally, the classification head utilized a Softmax function to transform the prediction scores of the backbone network into the classification probabilities.

CLAPE is a highly flexible framework, allowing customization of each essential component. In the loss computation module, one may employ different contrastive loss functions such as that proposed in DrLIM [31] or lifted structure loss [32], and other backbone models, such as MLP and RNN, were also suitable for use with CLAPE.

The pre-trained model was used as a feature extractor to avoid tedious manual feature extraction procedures. However, researchers may choose to fine-tune the pre-trained model, which has been shown to produce better performance but requires higher computational and time consumption [33] resembling the training scheme described in Supplementary Figure 1 available online at http://bib.oxfordjournals.org/. Furthermore, multiple pre-trained protein language models can be applied to the sequence embedding module [34].

### CLAPE-DB accurately predicted the DNA-binding sites with a better generalization ability

We evaluated the performance of the proposed CLAPE-DB (CLAPE DNA-binding) model on two protein–DNA datasets, as described in Table 1. To assess the performance of CLAPE-DB, we conducted experiments on both Dataset1 and Dataset2 using independent testing sets TE46 and TE129, respectively. We compared the results with existing DNA-binding sites prediction tools based on protein sequence input. CLAPE-DB outperformed other methods on both datasets (Table 2 and Table 3). Specifically, in TE46, CLAPE-DB trained on TR646 outperformed the second-best model DBPred [15] by a large margin, achieving a specificity of 0.835, a recall of 0.747, a precision of 0.306, an F1-score of 0.434, an MCC of 0.401 and an AUC of 0.871 in Dataset1 (Table 2), yielding a significant improvement over DBPred by 6.5%, 5.5%, 25.9%, 19.9%, 25.3% and 9.6%. Notably, DBPred used a manual feature extraction process, and a similar CNN model as CLAPE-DB, highlighting the advantages of using pre-trained models over handcrafted features.

**Table 1:** Summary of benchmark protein–DNA binding datasets

Datasets	Dataset1		Dataset2	
	TR646	TE46	TR573	TE129
DNA-binding residues	15 636	965	14 479	2240
Non-binding residues	298 503	9911	145 404	35 275
% of binding residues	4.98	8.87	9.06	5.97

**Table 2:** Comparison of CLAPE-DB with other sequence-based methods on TE46

Models	Spe	Rec	Pre	F1	MCC	AUC
DRNAPred	0.692	0.677	0.185	0.291	0.226	0.755
DNAPred	0.655	0.671	0.157	0.254	0.194	0.730
SVMnuc	0.666	0.668	0.154	0.250	0.192	0.715
NCBRPred	0.674	0.677	0.165	0.265	0.207	0.713
DBPred	0.784	0.708	0.243	0.362	0.320	0.794
**CLAPE-DB**	**0.835**	**0.747**	**0.306**	**0.434**	**0.401**	**0.871**

**Table 3:** Comparison of CLAPE-DB with other sequence-based methods on TE129

Models	Spe	Rec	Pre	F1	MCC	AUC
DRNAPred	0.937	0.233	0.190	0.210	0.155	0.693
DNAPred	0.954	0.396	0.353	0.373	0.332	0.845
SVMnuc	0.966	0.316	0.371	0.341	0.304	0.812
NCBRPred	**0.969**	0.312	0.392	0.347	0.313	0.823
**CLAPE-DB**	0.955	**0.464**	**0.396**	**0.427**	**0.389**	**0.881**

Moreover, we trained and evaluated CLAPE-DB on Dataset2, and compared it with other existing tools (Table 3). CLAPE-DB also achieved better predictive capability on this dataset. Dataset2 was proposed as a benchmark dataset for structure-based models, and we compared the metrics of several structure-based models (Supplementary Table 1 available online at http://bib.oxfordjournals.org/). Although CLAPE-DB did not incorporate any structure information, it outperformed the structure-based models such as COACH-D, NucBind and DNAbind. Notably, the GraphBind model used predicted protein structure exhibited a poor performance with an AUC of 0.816, lower than that of CLAPE-DB. The results suggested that structure-based models required accurate protein structure to achieve acceptable prediction results. Moreover, compared to the structure-based models, CLAPE-DB used only a pre-trained language model and a simple backbone network to process the data, which reduced the model complexity and enhanced the inference speed, while maintaining accuracy.

**Figure 2:**
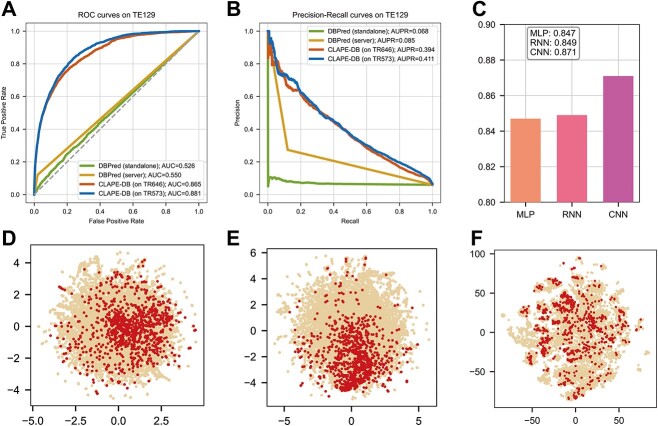
Evaluation of CLAPE-DB model performance. (**A**) Receiver operating characteristic (ROC) curves of DBPred and CLAPE-DB models. CLAPE-DB showed a larger area under ROC curve (AUC) than DBPred, indicating a better generalization ability. (**B**) Precision–recall (PR) curves of DBPred and CLAPE-DB models. (**C**) Comparison of different backbone models, where we used an LSTM model to represent RNN. (**D**) t-SNE dimension reduction plot of the first layer output of a randomly initialized 1DCNN model. (**E**) t-SNE dimension reduction plot of the first layer output of CLAPE-DB. (**F**) t-SNE dimension reduction plot of the original sequence features generated by ProtBert. All of (**C**–**F**) were tested and plotted using TE46, with cream-colored and red data points indicating non-binding sites and DNA-binding sites, respectively.

To test the generalization ability of CLAPE-DB, we trained a model on Dataset1 and tested it on Dataset2 (Figure 2A and B), and DBPred was also tested as the same strategy for a fair comparison. The prediction metrics of CLAPE-DB surpassed DBPred by a large margin, AUC and area under PR curve (AUPR) of CLAPE-DB were 0.865 and 0.394, respectively, while the metrics of DBPred standalone version were 0.526 and 0.068, which was slightly higher than a random choice result. Besides, the result of CLAPE-DB was merely lower than CLAPE-DB trained on TR573 (0.871 and 0.881, respectively), showing that our proposed model had a superior generalization ability compared to the DBPred model. For further clarification of the generalization ability of CLAPE-DB, we selected the dataset TE181 (Supplementary Table 2 available online at http://bib.oxfordjournals.org/) created by Yuan *et al.* [35], which is unseen in both Dataset 1 and Dataset2, and tested the performance of CLAPE-DB trained on TR573 for an impartial evaluation with existing models. CLAPE-DB still showed a better performance than other sequence-based models and most structure-based models (Supplementary Table 3 and Supplementary Table 4 available online at http://bib.oxfordjournals.org/). The data quality and quantity could vary across the datasets, which makes it hard to capture the latent distribution, and it is worth noting that the gains of model performance on Dataset1 are significantly higher than that on Dataset2 compared to other models, which further validates that our proposed model has superior generalization ability.

### Backbone network comparison and feature visualization of CLAPE-DB

We compared the performance of different mainstream backbone networks, including MLP, RNN and CNN, and we used an LSTM model to represent the RNN model. 1DCNN model achieved the best performance among the three commonly used models (Figure 2C). Although RNN was specifically designed for sequence modeling tasks, our finding suggested that the CNN was more suitable for predicting DNA-binding sites. This might be because RNN models process sequential data from left to right, whereas DNA-binding residues are predominately determined by spatial structures rather than simple sequential order. While CNN models the protein sequences using sliding windows, which incorporate relative positional information of amino acids inherently, amino acids are treated as independent tokens in RNN models [36].

We compared the embedding space generated by the CLAPE-DB model and an untrained, randomly initialized 1DCNN model, and utilized *t*-SNE (*t*-distributed Stochastic Neighbor Embedding) dimension reduction method. Our results showed that CLAPE-DB learned a discriminative embedding space, while the data points were randomly distributed in the space after being processed by the untrained model (Figure 2D and E). Moreover, CLAPE-DB was able to effectively distinguish the DNA-binding and non-binding samples in the embedding space of each layer, with the distinction becoming more pronounced as the convolutional layer approached the output layer (Supplementary Figure 2 available online at http://bib.oxfordjournals.org/). In addition, we plotted the dimension reduction result of the raw features generated by ProtBert, which showed that the raw features were not well separated before model processing. Our results showed that CLAPE-DB was effective at distinguishing data samples from different classes (Figure 2F).

### Contrastive learning improved the model performance

In the loss computation module, CLAPE-DB utilized a combination of triplet center loss (TCL) [37] and class-balanced focal loss [38, 39]. To analyze the effectiveness of the loss functions, we performed ablation studies. TCL and focal loss generated discriminative embeddings in high-dimensional space, and both loss functions led to better performance than the commonly used cross-entropy loss (Table 4). In addition, the model performance decreased when solely applying classification or contrastive loss (Table 4). Therefore, the joint loss is focal loss guided by the triplet center loss. Furthermore, the improvement in the AUPR value indicated that class-balanced focal loss and contrastive learning methods showed a better ability to cope with imbalanced datasets.

**Table 4:** Model performance using different loss functions

Loss functions	AUC	AUPR
Cross-entropy	0.849	0.438
Cross-entropy + TCL	0.861	0.445
Focal loss	0.865	0.459
**Focal loss + TCL**	**0.871**	**0.463**

We also visualized the embeddings generated by the first layer using focal loss only and jointly using focal loss and TCL. As expected, though the embeddings of DNA-binding sites and non-binding sites separated to a certain extent, the embeddings generated by joint loss functions showed a single clustering center, and the positive and negative samples were more discriminative (Supplementary Figure 3 available online at http://bib.oxfordjournals.org/). The single and uniform cluster center could benefit the classification performance according to the previous studies [37, 40].

### Parameter impact of loss functions

**Figure 3:**
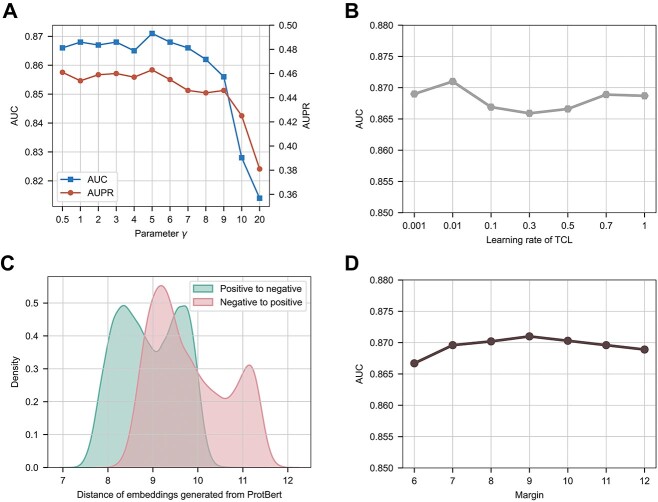
Hyperparameter optimization of loss functions. (**A**) Trends in the AUC and AUPR metrics with varying parameter $\gamma$. Both metrics reached their maximum values when $\gamma$ was set to 5. (**B**) Trend in the AUC metric with varying learning rate of the triplet center loss (TCL). The AUC reached its maximum value when the learning rate was set to 0.01. (**C**) Distance distribution of negative and positive samples, where the distance was defined as the maximum Euclidean distance between a given sample and the sample from the opposite class. The embedding used to calculate the distance was the raw sequence embedding generated from ProtBert. (**D**) The trend in the AUC metric with varying margin of TCL. The AUC reached its maximum value when the margin was set to 9.

The hyperparameters were utilized in TCL and class-balanced loss matter in model training and inference; therefore, we analyzed and adjusted the hyperparameters in the loss functions. We adjusted the hyperparameter $\gamma$ in class-balanced focal loss from 1 to 10 and observed the AUC and AUPR values remained relatively stable within a specific range, but with an increase of $\gamma$, both metrics displayed a significant decline (Figure 3A). To verify our findings, we conducted further tests with $\gamma$ values of 0.5 and 20. Finally, we adopted a $\gamma$ value of 5.

The cluster centers in TCL were randomly initialized, and we tested the model performance by adjusting the parameter learning rate and margin (m). Previous studies suggested that the learning rate for optimizing the cluster center should be relatively large [40]. However, we found that the AUC value was the highest when the learning rate was set to a relatively small value of 0.01 (Figure 3B). The margin was another crucial hyperparameter in TCL, and we intuitively visualized the distance distribution of TR646 to guide our choice of parameter *m*. The distances from negative to positive and positive to negative were distributed from 7 to 12 (Figure 3C). Thus, we adjusted the margin value based on the distribution plot. We found that the AUC was maximized when the margin was set to 9, which was consistent with our expectations (Figure 3D).

### CLAPE-DB captured the properties distribution of amino acids

It is widely acknowledged that protein–DNA binding preferences are reflected in the sequences and structures of proteins and DNA [41]. For instance, proteins can bind DNA modules via hydrogen bonds and hydrophobic interactions. Such biological phenomena are related to the amino acid composition and properties of proteins.

**Figure 4:**
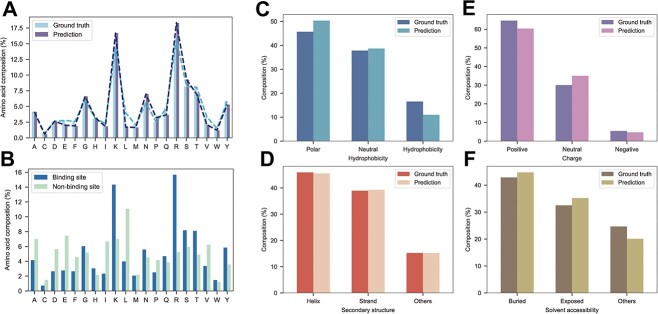
Analysis of amino acid composition and properties. (**A**) Distribution of amino acid composition in DNA-binding sites and non-binding sites. (**B**) Comparison of the distribution of experimental DNA-binding sites with predicted binding sites. (**C**–**F**) Comparison of the distribution of amino acid physicochemical properties and structural properties of real DNA-binding sites with predicted binding sites. (**C**–**F**) represents hydrophobicity, secondary structure, charge and solvent accessibility, respectively.

To this end, we performed a statistical analysis of the amino acid composition of DNA-binding sites and non-binding sites using the TE129 dataset. Lysine, arginine and tyrosine were the predominant amino acid types in the DNA binding sites, while alanine and leucine were the primary amino acid types in the non-binding sites (Figure 4A). Furthermore, we compared the amino acid type distribution of predicted results and the ground truth (Figure 4B) and used the Kullback–Leibler (KL) divergence to measure the distance of discrete distributions. The shapes of distributions of prediction and ground truth were quite similar, and the forward and reverse KL divergence were 0.024 and 0.028, respectively, which were close to 0, indicating that the two distributions were semblable. Our results demonstrated that CLAPE-DB could accurately capture the amino acid composition features of DNA-binding residues.

In addition, we analyzed the physicochemical properties of amino acids by extracting features from protein sequence and structure, and subsequently tested several selected properties, such as hydrophobicity, charge, secondary structure and solvent accessibility. The t-SNE dimension reduction visualization revealed that different types of amino acid physiochemical properties were segregated into various clusters (Supplementary Figure 4A–D available online at http://bib.oxfordjournals.org/). Our results illustrated that the large-scale pre-trained protein language model ProtBert was capable of effectively learning the properties of amino acids. Such models were identified as appropriate feature extractors to replace handcrafted descriptors, which is congruent with previous studies [24].

Moreover, CLAPE-DB was proved successful in predicting not only the distribution of amino acids but also their properties. The binding sites predicted by CLAPE-DB exhibited a similar composition of different properties to the real DNA-binding sites (Figure 4C–F).

### Comparative and empirical case study

**Figure 5:**
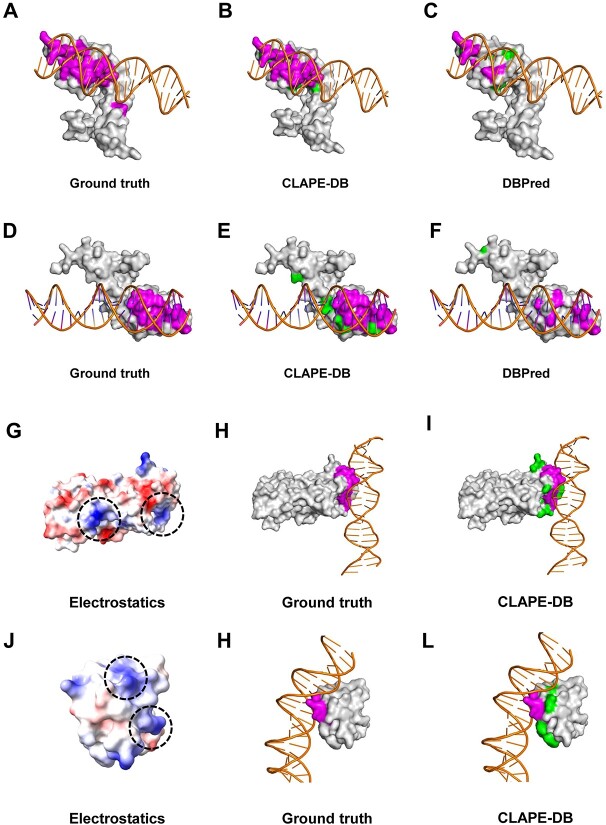
Comparative and empirical case studies. (**A**–**C**) Analysis of the DNA-binding sites for protein 5H3R_A, where (**A**) represents the experimental result, (**B**) and (**C**) represent the results predicted by CLAPE-DB and DBPred, respectively. (**D**–**F**) Analysis of the DNA-binding sites for protein 6C2S_A, where (**D**) represents the experimental result, (**E**) and (**F**) represent the results predicted by CLAPE-DB and DBPred, respectively. Magenta residues indicate the experimental binding sites and true positives predicted by the models, green residues indicate the false positives generated by the models and gray residues indicate non-binding residues. Orange double-helix structures represent DNA molecules. (**G**–**H**) Comparison of the surface charge distribution and DNA-binding sites for protein 5GPC_A between the experimental and CLAPE-DB predicted results. (**J**–**L**) Comparison of the surface charge distribution and DNA-binding sites for protein 5J2Y_A between the experimental and CLAPE-DB predicted results. Blue and red residues indicate positively and negatively charged residues, respectively. The dashed circles in (**G**) and (**I**) indicate the human-predicted DNA-binding sites based on electrostatics.

To intuitively visualize and compare the prediction performance of DNA-binding residues of CLAPE-DB, we selected two protein structures for illustration purposes: multiple antibody resistance regulator (MarR) families (PDB ID: 5H3R, chain A, denoted as 5H3R_A) and transcription repressor protein CouR (PDB ID: 6C2S, chain A, denoted as 6C2S_A). CLAPE-DB made an accurate prediction of DNA-binding sites, while DBPred only captured a limited number of true positive sites, highlighting the superior prediction ability of CLAPE-DB. In addition, the majority of false-positive sites were located in close proximity to binding sites (Figure 5A–F). Our results suggested that CLAPE-DB effectively learned the amino acid properties that were spatially adjacent and the structural information without relying on protein structures.

DNA molecules are negatively charged and tend to bind the positively charged regions of proteins. The structure of the protein–DNA binding area could be divided into several domains with specific patterns [42]. Empirical observations and computational properties can be utilized to infer the DNA-binding sites from the protein structure. However, such methods have significant limitations. Firstly, some proteins, such as intrinsically disordered proteins, are unstructured when not bound by ligands like DNA [43]. Secondly, the inferred probable DNA-binding sites using the surface charge distribution and protein structure are often quite different from the real binding sites. To illustrate the limitations of empirical analysis, we selected two protein structures: the transcription regulatory protein FadR (PDB ID: 5GPC, chain A, denoted as 5GPC_A) and bacteria quorum-sensing repressor protein RsaL (PDB ID: 5J2Y, chain A, denoted as 5J2Y_A). In both protein structures, multiple possible binding sites were identified based on the charge distribution (Figure 5G and J), and it was difficult to determine which part of the protein would bind the major or minor groove of DNA. However, CLAPE-DB precisely distinguished the binding sites, and the false-positive sites were not influenced by the other positively charged locations (Figure 5H and I and Figure 5K and L). It should be noted that the empirical binding site identification relied on the experimental structures, which was limited when lacking protein structures or using inaccurately predicted structures.

### CLAPE was a general ligand-binding sites prediction framework

**Figure 6:**
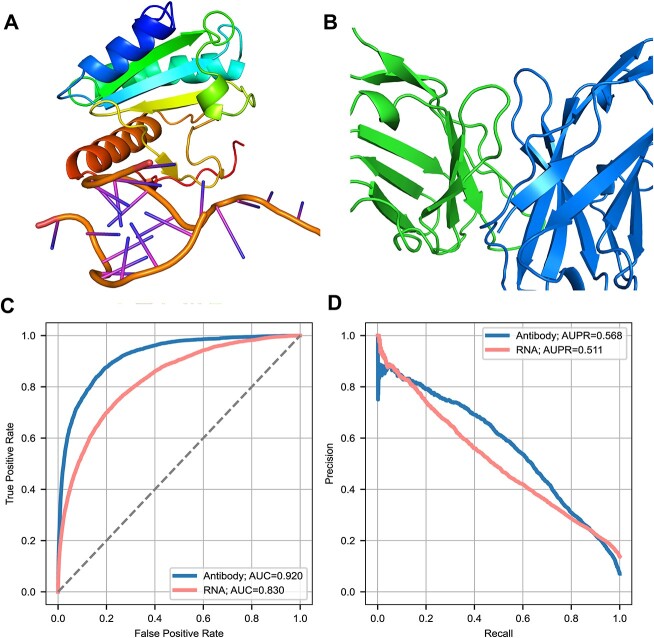
General binding sites prediction ability of CLAPE. (**A**–**B**) Binding diagrams of protein–RNA (PDB ID: 5GAN) and antibody–antigen (PDB ID: 1OAY), demonstrating the ability of CLAPE to predict protein–ligand binding sites. (**C**–**D**) ROC and PR curves of CLAPE-RB and CLAPE-AB models. CLAPE-RB achieved an AUC of 0.830 and an AUPR of 0.511, while CLAPE-AB achieved an AUC of 0.920 and an AUPR of 0.568.

CLAPE could serve as a general framework for predicting other ligand-binding sites, including protein–RNA and antibody–antigen binding sites. (Figure 6A and B). We collected benchmark datasets of protein–RNA and antibody–antigen binding sites (Supplementary Table 5 available online at http://bib.oxfordjournals.org/) and trained CLAPE on these datasets. The resulting models were denoted as CLAPE-RB (CLAPE RNA-binding) and CLAPE-AB (CLAPE-Antibody). Both CLAPE-RB and CLAPE-AB performed well on the testing sets, with CLAPE-AB achieving the AUC of 0.920 (Supplementary Table 6 available online at http://bib.oxfordjournals.org/), which was relatively high and could be applied to accurately predict the paratope of a given antibody sequence. Moreover, the AUC of CLAPE-RB trained on TE161 was 0.830 (Supplementary Table 6 available online at http://bib.oxfordjournals.org/), which surpassed the existing sequence-based RNA-binding sites models [44, 45]. We also plotted the ROC and AUC curves to visualize the overall model performance of CLAPE-RB and CLAPE-AB (Figure 6C and D).

Furthermore, we trained CLAPE-RB on a separate protein–RNA dataset comprising TR495 and TE117 (Supplementary Table 7 available online at http://bib.oxfordjournals.org/), which were widely used benchmarks for structure-based models. CLAPE-RB outperformed existing sequence-based models in predicting RNA-binding sites on TE117. While the performance of CLAPE-RB was marginally lower than that of the structure-based model GraphBind, it performed better than Nucleic, a CNN model predicting RNA-binding sites based on grids of the protein surface (Supplementary Table 8 available online at http://bib.oxfordjournals.org/). Similarly, CLAPE-RB outperformed GraphBind based on inaccurately predicted protein structure, which highlighted the potential of CLAPE to overcome the limitations of structure-based models. Our results indicated that CLAPE was a versatile framework that could predict ligand-binding sites of a given protein sequence for a range of ligands. Furthermore, our experiments demonstrated that CLAPE was an effective predictor of ligand-binding sites, even in the absence of structural information, achieving relatively high performance.

## DISCUSSION

Protein–DNA binding plays an essential role in many life activities, and studies on the binding properties contribute to the understanding of genome transcription and regulation. Accurate identification of DNA-binding sites of proteins is a crucial step in modeling the protein–DNA interactions. Various models have been developed using machine learning and deep learning techniques to identify DNA-binding sites from protein sequence or structure [15, 23]. However, current tools rely on tedious manual feature extraction processing, which is time consuming and redundant. In addition, the accuracy of sequence-based models still needs to be increased, and the performance of the structure-based models is affected mainly by the accuracy of protein structure. Given these limitations, it is imperative to develop a satisfactory sequence-based model that utilizes protein sequence information alone to predict DNA-binding sites. To address the existing challenges and improve the performance of the sequence-based models, we proposed CLAPE, a deep learning framework that combines a large-scale pre-trained protein language model and contrastive learning technique to predict DNA-binding sites of a given protein sequence accurately.

In this study, we presented the overall architecture of CLAPE, which was composed of three main components. Firstly, we utilized a pre-trained model, ProtBert, without fine-tuning, to conduct feature extraction. Secondly, we employed a 1DCNN to process the sequence feature and generate the classification score. Finally, we jointly optimized a class-balanced focal loss and a contrastive triplet center loss to address the issue of imbalanced data, which resulted in a more discriminative embedding space with a single cluster center.

The proposed CLAPE-DB model for predicting DNA-binding sites demonstrated superior performance compared to existing sequence-based models on two benchmark datasets, as indicated by all metrics, with an AUC of 0.871 and 0.881, respectively. Furthermore, in cases where accurate protein crystal structures were unavailable, CLAPE-DB outperformed structure-based models by a large margin. In addition, we evaluated the generalization ability of the CLAPE-DB model on independent datasets and found that CLAPE-DB exhibited better generalization performance than the second-best model, DBPred. These results suggested that CLAPE-DB effectively learned the underlying latent distribution of DNA-binding sites.

To mitigate the effects of imbalanced data, we implemented the class-balanced focal loss in our proposed CLAPE model. There were several augmentation approaches from the aspect of the dataset, such as [46] (SMOTE) to interpolate new data in the embedding space. We also tested several data augmentation strategies, including SMOTE, nearest neighbor and random noise methods on Dataset1 (Supplementary Table 9 available online at http://bib.oxfordjournals.org/). Furthermore, incorporating the newly solved protein–DNA complexes into the dataset could enhance the prediction performance and generalization ability of the model.

Our study demonstrated that a large-scale pre-trained protein language model could extract protein sequence features effectively, eliminating the need for designing handcrafted features. In this study, we only evaluated the ProtBert as the feature extractor, but other pre-trained protein models such as RITA [47] and ESM-2 [48], as reviewed in detail by Hu *et al*. [34], could be used for feature generation. Here, we tested the performance of CLAPE-DB applying a larger protein language model ESM-2 as the feature extractor, which contained more parameters than ProtBert, and the model performance of CLAPE-DB was improved using ESM-2, which was consistent with our expectation (Supplementary Table 10 available online at http://bib.oxfordjournals.org/). Furthermore, fine-tuning the model is also a choice under sufficient computational resources. Here, we fine-tuned the last three layers of the ESM-2 model and obtained a better performance in various metrics (Supplementary Table 11 available online at http://bib.oxfordjournals.org/). As mentioned above, various contrastive losses can be applied to CLAPE including both supervised loss, such as triplet center loss used in our work, and unsupervised loss such as InfoNCE. We evaluated the model performance under the InfoNCE loss, which is comparable with TCL (Supplementary Table 12 available online at http://bib.oxfordjournals.org/). To be pointed out, we picked TCL as one of the possible contrastive loss functions to illustrate that the proposed CLAPE model is a general protein–ligand binding sites prediction model, and various contrastive losses may lead to superior and inferior performance compared with TCL.

Although the CLAPE-DB was designed for sequence-based prediction tasks, it is possible to use the features generated by the pre-trained model for the structure-based model, as demonstrated in related studies [49]. In addition, we conclude that CLAPE is a general prediction framework for identifying ligand-binding sites of a given protein sequence based on the results of our experiments.

Overall, the deep learning model CLAPE proposed in our study achieved high performances in predicting both DNA- and ligand-binding sites by combining pre-trained models with contrastive learning methods. The promising and general framework can be applied in future studies to facilitate protein function annotation, protein engineering and drug discovery.

## METHODS

### Dataset description

In this study, we evaluated and compared the performance of our proposed model, CLAPE, with existing classifiers using two widely used benchmark datasets, denoted as Dataset1 and Dataset2. The training and testing datasets were denoted as TR and TE, respectively. Both datasets were preprocessed by similar procedures to improve the robustness of models and avoid bias due to the imbalanced data distribution, such as reducing the sequence similarity using a cutoff of 30% with CD-HIT [50]. The binding sites were defined similarly in both datasets as residues with a distance less than 0.5 plus the sum of the Van der Waals radius of the two nearest atoms between the residue and the nucleic acid molecule. Table 1 provides a summary of the benchmark datasets, and the details of both datasets are described below.

Dataset1 was introduced by the study of the DBPred model, a sequence-based deep learning method for predicting DNA-binding residues [15]. The dataset was composed of 646 proteins as the training set (TR646) with 15 636 DNA-binding sites and 298 503 non-binding sites, and 46 proteins as the testing set (TE46) with 956 DNA-binding sites and 9911 non-binding sites.

Dataset2 was originally proposed by the study of GraphBind, a structure-based GNN model for identifying nucleic-acid-binding residues [23]. This dataset consisted of protein–DNA complex structural data extracted from the BioLiP database [51], with 573 proteins as a training set (TR573) with 14 479 DNA-binding residues and 145 404 non-binding residues, and 129 proteins as a testing set (TE129) with 2240 DNA-binding residues and 35 275 non-binding residues. GraphBind employed a data augmentation approach on the training set to alleviate the impact of the data imbalanced issue, hence we used the same augmented data annotations as GraphBind.

To assess the prediction capability of our proposed model CLAPE on diverse ligand-binding sites, we gathered three different datasets comprising protein–RNA and antibody–antigen interactions. The protein–RNA datasets were created by Xia *et al*. based on the GraphBind model, and Patiyal *et al*. [45], based on the pprint2 model. The antibody–antigen dataset was collected from the SAbDab database[52] . To ensure a fair comparison with existing models, we applied the same data preprocessing procedure as used for defining DNA-binding sites.

### Protein sequence embedding

The protein sequences were first input into ProtBert [30], a pre-trained model, to generate high-dimensional embeddings. ProtBert is a member of the ProtTrans family of pre-trained models and is based on the BERT architecture. The ProtTrans models were trained on large-scale protein sequences and have been commonly used for predicting protein structure and properties. The dimension of the protein embedding generated by ProtBert was 1024. It is important to note that ProtBert was not fine-tuned during subsequent training steps, and the sequence embedding process was performed using HuggingFace’s Transformers Python package [53].

### Backbone 1DCNN model and classification head

We utilized a one-dimensional convolutional neural network (IDCNN) as our backbone model to obtain a residue-level classification score. To maintain the same length of input and output protein sequence and obtain a unified token-level classification result, we applied padding for different convolutional kernel sizes. The stride of every layer was set to 1, and we utilized rectified linear unit as an activation function to introduce nonlinearity to the model. We applied dropout and batch normalization techniques to enhance the robustness and generalization ability of the model. Our CLAPE-DB model consisted of four 1DCNN layers as the backbone model. The raw dimension was 1024, and the output dimension of the four layers were 1024, 128, 64 and 2, respectively. The classification head part contained a Softmax function to scale the output value between 0 and 1 as a mutually exclusive prediction score, representing the classification probability of DNA-binding sites.

### Binary classification loss function

We applied a class-balanced focal loss to address the data imbalance issue. The focal loss was introduced by Lin *et al*. [38] and places more emphasis on classes with fewer samples in the loss function. It also considers the difficulty of samples based on the classification probability provided by the classifier. The focal loss is formulated as follows:


(1) \begin{equation*} \mathrm{FL}\left({p}_t\right)=-{\mathrm{\alpha}}_t{\left(1-{p}_t\right)}^{\mathrm{\gamma}}\log \left({p}_t\right) \end{equation*}

where ${p}_t$ is the classification probability of a particular class, $1-{p}_t$ is the modulator and $\gamma$ is a hyperparameter to adjust the weight of hard and easy samples. In the original paper, $\mathrm{\alpha}$ is also a parameter to give the weight of minority and majority samples, which is influenced by $\gamma$. We applied an effective number to reweight the focal loss, which was proposed by Cui *et al*. [39]. Effective number was proposed to model the real space covered by all samples, which could be used as a weight for imbalanced data. The class-balanced focal loss can be formulated as


(2) \begin{equation*} {\displaystyle \begin{array}{c}{L}_{\mathrm{focal}}=-\frac{1-\beta }{1-{\beta}^{n_y}}{\sum}_{i=1}^C{\left(1-{p}_i^t\right)}^{\mathrm{\gamma}}\log \left({p}_i^t\right)\end{array}} \end{equation*}

where ${E}_n=\left(1-{\beta}^n\right)/\left(1-\beta \right)$ refers to the effective number of the class; we set $\beta$ to 0.999 in our study according to Cui *et al*. [39]. The class-balanced focal loss was jointly optimized with contrastive loss, as described in the following parts.

### Contrastive learning loss

We applied a contrastive loss named triplet center loss (TCL) [37], which is a supervised approach that takes into account the labels of the training data, which finds a high-dimensional embeddings space that represents residues, which makes the DNA-binding residues and non-binding residues far away from each other and forces the residues of different classes to be close to respective cluster centers. Here, the cluster center is learnable and was randomly initialized at the beginning of the training process. The formulation of TCL can be mathematically expressed as follows:


(3) \begin{equation*} {\displaystyle \begin{array}{c}{L}_{tc}=\sum_{i=1}^M\max \left(D\left({f}_i,{c}_{y^i}\right)+m-\underset{j\ne{y}^i}{\min }D\left({f}_i,{c}_j\right),0\right)\end{array}} \end{equation*}

where ${c}_{y_i}$ is the center of the given class ${y}_i$, and ${f}_i$ refers to the classification probability predicted by the model. $D$ indicates the Euclidean distance between residue embeddings: $D\left({f}_i,{c}_{y^i}\right)=\frac{1}{2}{\left|{f}_i-{c}_{y^i}\right|}_2^2$. The total loss was weighted by class-balanced focal loss and TCL using a hyperparameter $\lambda$, which was set to 0.1 in our study after searching, and the loss function could be formulated as follows:


(4) \begin{equation*} {\displaystyle \begin{array}{c}L={L}_{\mathrm{focal}}+\mathrm{\lambda} {L}_{tc}\end{array}} \end{equation*}

The backpropagation stops at the embedding generated by ProtBert, which means we did not fine-tune the pre-trained language model.

### Evaluation metrics

In this study, we employed several classification evaluation metrics to ensure consistency with the previous studies. The threshold-dependent metrics included specificity (Spe), precision (Pre), recall (Rec), F1-score and Matthews correlation coefficient (MCC). The metrics can be formulated as follows:


(5) \begin{equation*} {\displaystyle \begin{array}{c}\mathrm{Spe}=\displaystyle\frac{\mathrm{TN}}{\mathrm{TN}+\mathrm{FP}}\end{array}} \end{equation*}

(6) \begin{equation*} {\displaystyle \begin{array}{c}\mathrm{Pre}=\displaystyle\frac{\mathrm{TP}}{\mathrm{TP}+\mathrm{FP}}\end{array}} \end{equation*}

(7) \begin{equation*} {\displaystyle \begin{array}{c}\mathrm{Rec}=\displaystyle\frac{\mathrm{TP}}{\mathrm{TP}+\mathrm{FN}}\end{array}} \end{equation*}

(8) \begin{equation*} {\displaystyle \begin{array}{c}\mathrm{F}1=2\times \displaystyle\frac{\mathrm{Pre}\times \mathrm{Rec}}{\mathrm{Pre}+\mathrm{Rec}}\end{array}} \end{equation*}

(9) \begin{equation*} {\displaystyle \begin{array}{c}\mathrm{MCC}=\displaystyle\frac{\mathrm{TP}\times \mathrm{TN}-\mathrm{FN}\times \mathrm{FP}}{\sqrt{\left(\mathrm{TP}+\mathrm{FP}\right)\times \left(\mathrm{TP}+\mathrm{FN}\right)\times \left(\mathrm{TN}+\mathrm{FP}\right)\times \left(\mathrm{TN}+\mathrm{FN}\right)}}\end{array}} \end{equation*}

where TP, FP, TN and FN stand for true positive (number of residues that are correctly classified as DNA-binding sites), false positive (number of residues that are incorrectly classified as DNA-binding sites), true negative (number of residues that correctly classified as non-binding sites) and false negative (number of residues that incorrectly classified as non-binding sites), respectively. Specifically, specificity indicates the portion of correctly predicted non-binding sites, precision measures the accuracy of residues predicted as DNA-binding sites, recall measures the portion of DNA-binding residues successfully discovered by the model, and F1-score is the harmonic mean of precision and recall. MCC evaluates the prediction ability of both positive and negative classes of the model and is commonly used in imbalanced data. Besides, we plotted the receiver operating characteristic (ROC) curve and precision–recall curve to illustrate the overall performance of a model and used two threshold-independent metrics area under ROC curve and area under PR curve as numerical evaluations of both curves.

**Key Points**

- Existing models are limited by tedious handcrafted feature extraction processing or high demand for computational resources.
- CLAPE-DB outperformed existing sequence-based models with a better generalization ability.
- CLAPE is a general model for predicting protein–ligand binding sites based on pre-trained protein language model and contrastive learning.

## Supplementary Material

supfig1_bbad488

supfig2_bbad488

supfig3_bbad488

supfig4_bbad488

manuscript_bib_supplementary_tables_bbad488

## Data Availability

The datasets of our study and the codes of CLAPE are freely available at https://github.com/YAndrewL/clape.

## Author Biographies

**Yufan Liu** is a Master student in the School of Pharmaceutical Sciences at Tsinghua University. His research interests include bioinformatics and deep learning.

**Boxue Tian** is currently an assistant professor in the School of Pharmaceutical Sciences at Tsinghua University, focusing on computational chemistry and biology, including the development and applications of computational algorithms to important biological systems and pharmaceutical sciences.
