# Supplementary figures and images for: Protein–DNA binding sites prediction based on pre-trained protein language model and contrastive learning
Source: Brief Bioinform. 2024 Jan 3;25(1):bbad488. doi: 10.1093/bib/bbad488 (PMC10782905; doi:10.1093/bib/bbad488)

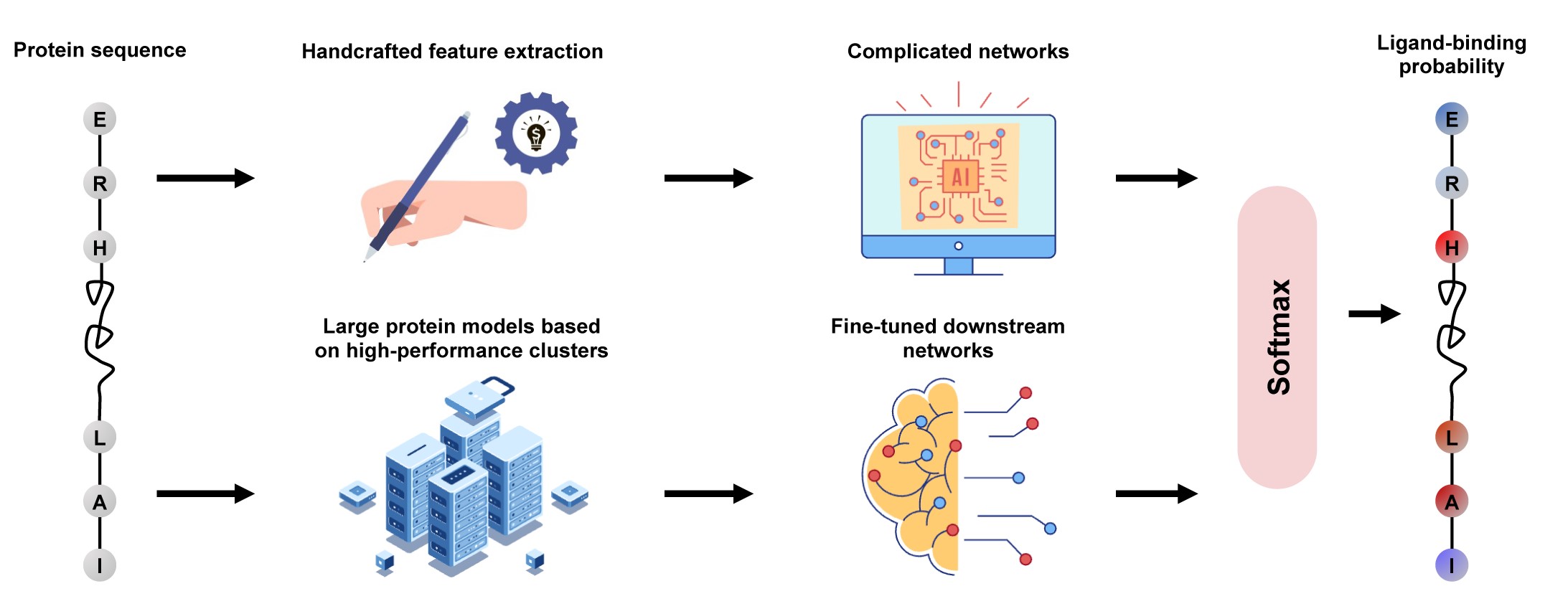

Supplement: supfig1_bbad488 [file supfig1_bbad488.jpeg]

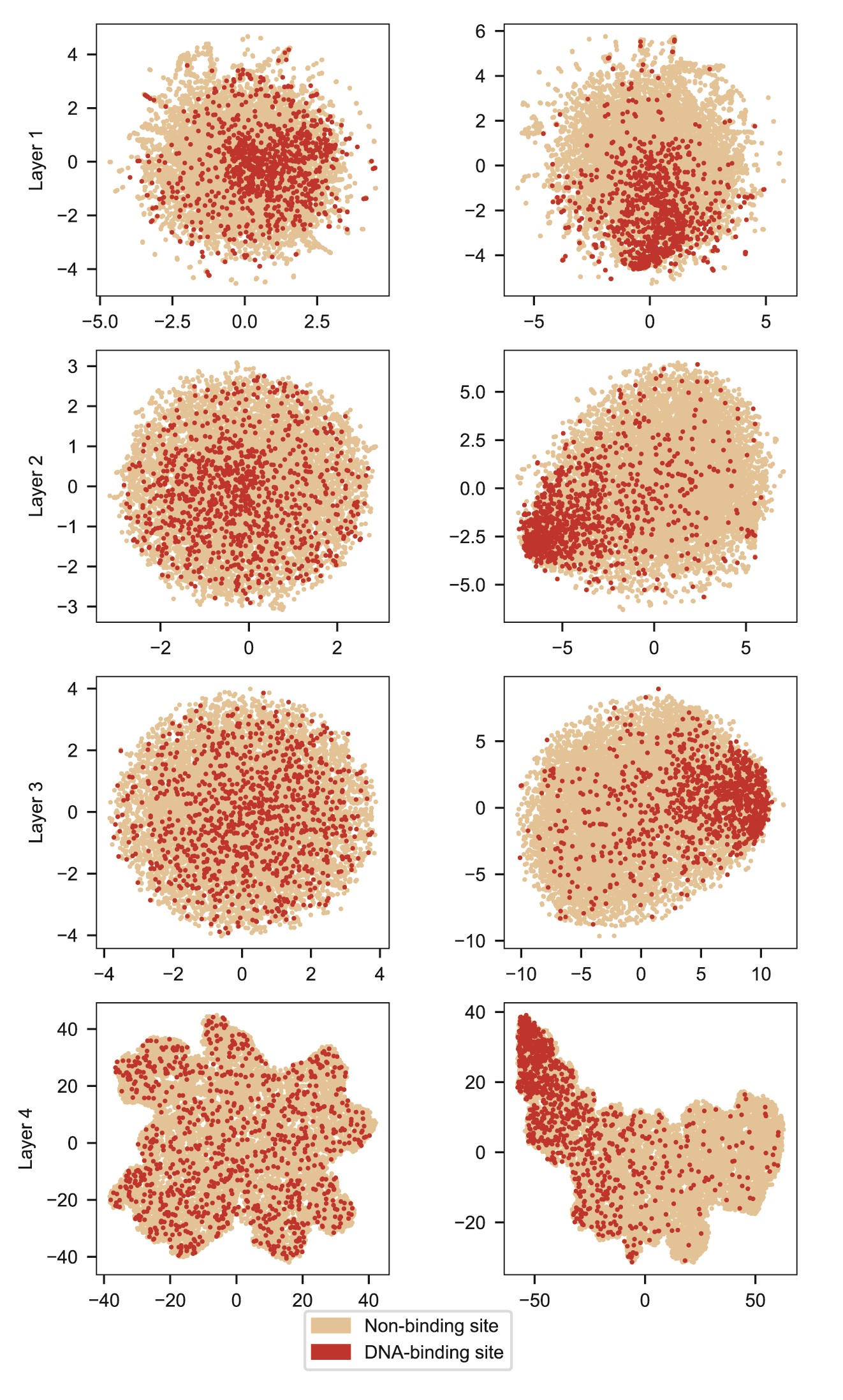

Supplement: supfig2_bbad488 [file supfig2_bbad488.jpeg]

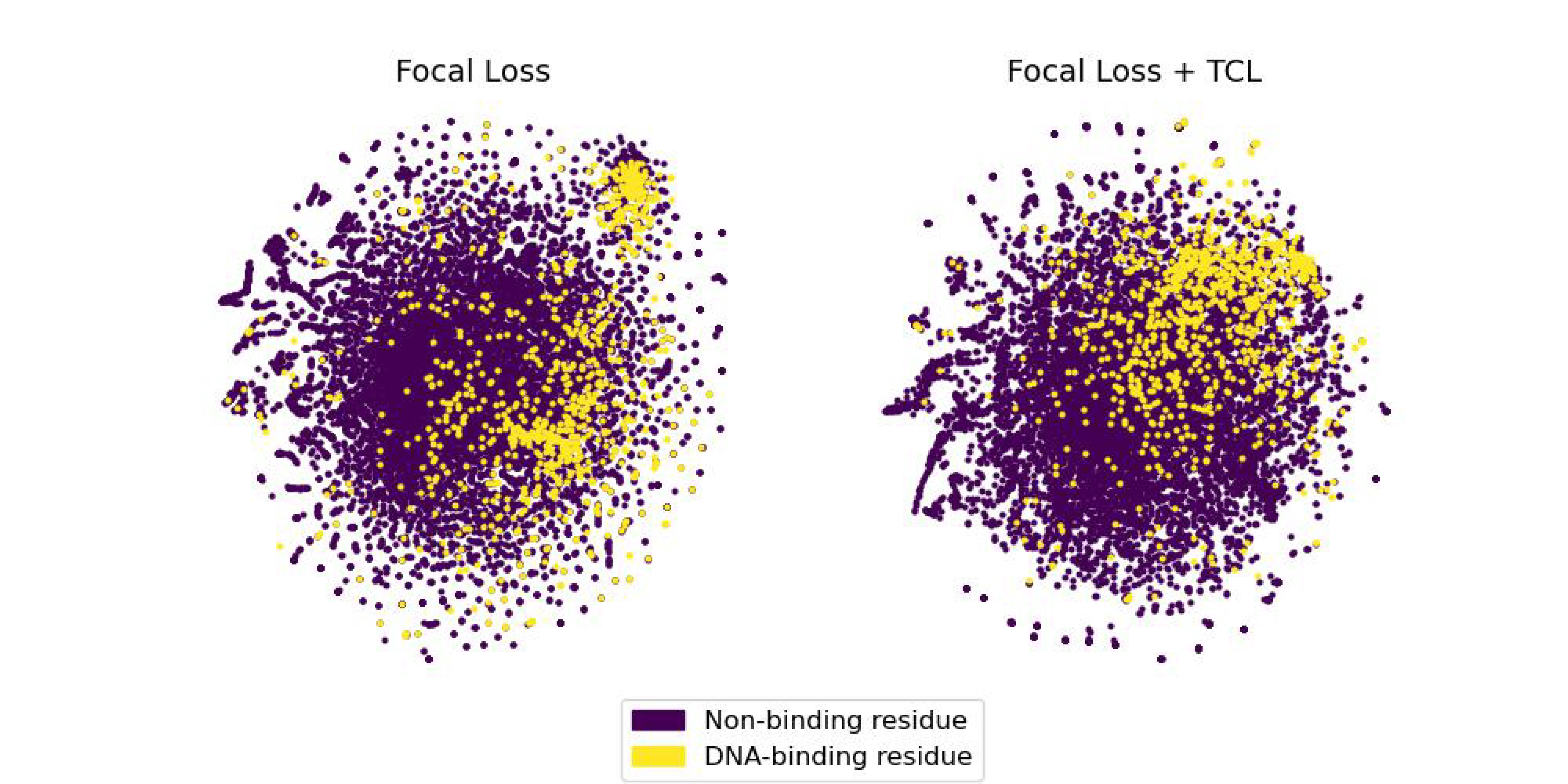

Supplement: supfig3_bbad488 [file supfig3_bbad488.jpeg]

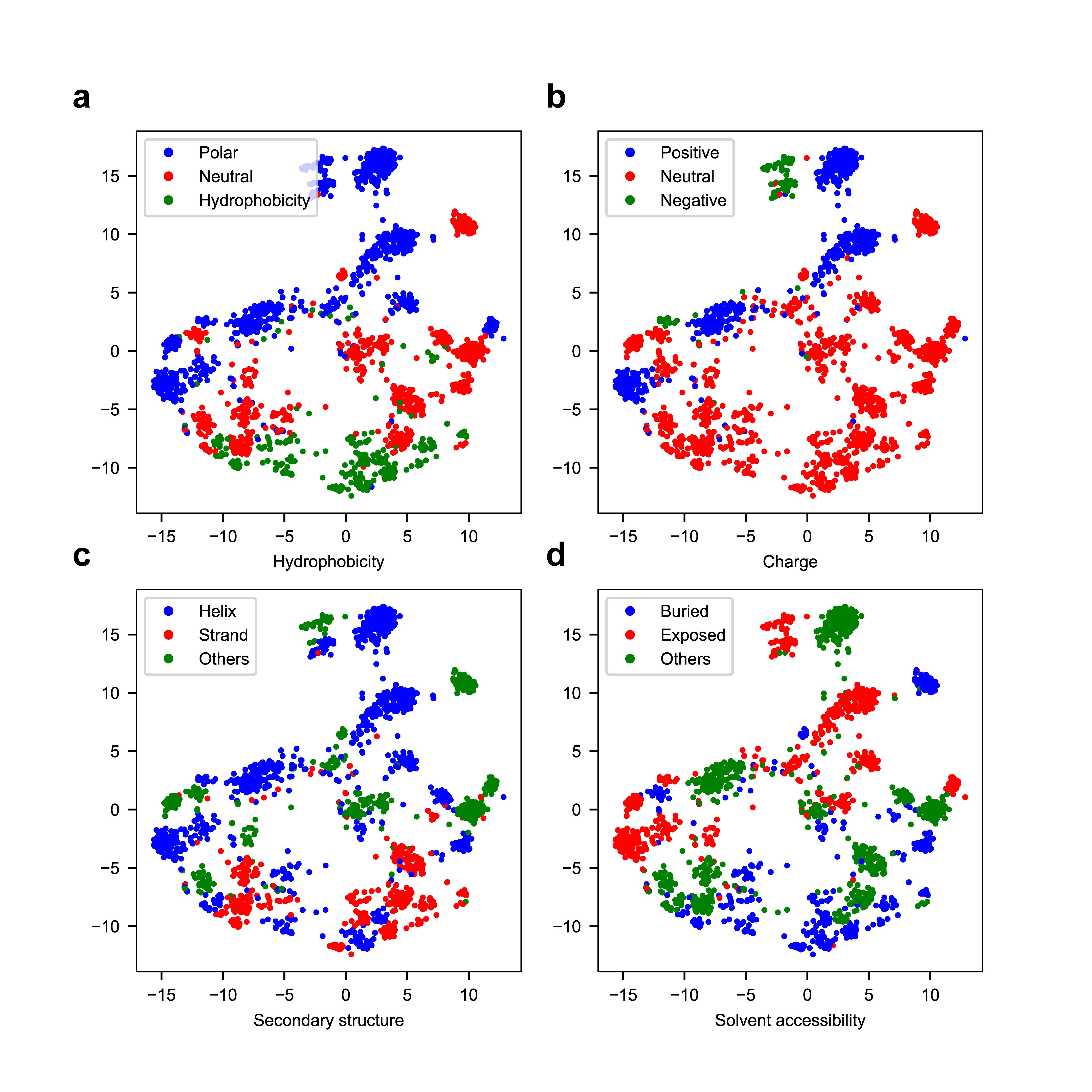

Supplement: supfig4_bbad488 [file supfig4_bbad488.jpeg]
