# Supplementary material for: Protein–DNA binding sites prediction based on pre-trained protein language model and contrastive learning
Source: Brief Bioinform. 2024 Jan 3;25(1):bbad488. doi: 10.1093/bib/bbad488 (PMC10782905; doi:10.1093/bib/bbad488)
Supplement: manuscript_bib_supplementary_tables_bbad488 [file manuscript_bib_supplementary_tables_bbad488.docx]

## Supplementary T**ables**

Supplementary Table 1: Comparison of structure-based model with other methods on TE129

| Models | Spe | Rec | Pre | F1 | MCC | AUC |
| --- | --- | --- | --- | --- | --- | --- |
| COACH-D | 0.958 | 0.367 | 0.357 | 0.362 | 0.321 | 0.710 |
| NucBind | 0.966 | 0.330 | 0.381 | 0.354 | 0.317 | 0.811 |
| DNABind | 0.926 | 0.601 | 0.346 | 0.440 | 0.411 | 0.858 |
| GraphBind^a^ | - | 0.439 | 0.310 | 0.362 | 0.320 | 0.816 |
| **GraphBind^b^** | **0.941** | **0.684** | **0.422** | **0.522** | **0.500** | **0.928** |

^a^ indicates results using predicted protein structure ^b^ indicates results using experimental protein structures. Results were obtained from Xia et al[^23^](#_ENREF_23)

Supplementary Table 2: Summary of protein-DNA binding sites dataset TE181

| Dataset | TE181 |
| --- | --- |
| DNA-binding residues | 3208 |
| Non-binding residues | 72050 |
| % of binding residues | 4.26 |

Supplementary Table 3: Comparison of CLAPE-DB with other methods on TE181

| Models | Spe | Rec | Pre | F1 | MCC | AUC |
| --- | --- | --- | --- | --- | --- | --- |
| DNAPred | 0.948 | 0.334 | 0.223 | 0.267 | 0.233 | 0.802 |
| SVMnuc | 0.960 | 0.289 | 0.242 | 0.263 | 0.229 | 0.803 |
| NCBRPred | **0.964** | 0.259 | 0.241 | 0.250 | 0.215 | 0.771 |
| **CLAPE-DB** | 0.931 | **0.413** | **0.212** | **0.280** | **0.252** | **0.824** |

Supplementary Table 4: Comparison of structure-based model with other methods on TE181

| Models | Spe | Rec | Pre | F1 | MCC | AUC |
| --- | --- | --- | --- | --- | --- | --- |
| COACH-D | **0.971** | 0.254 | 0.280 | 0.266 | 0.235 | 0.655 |
| NucBind | 0.960 | 0.293 | 0.248 | 0.269 | 0.234 | 0.796 |
| DNABind | 0.904 | 0.535 | 0.199 | 0.290 | 0.279 | 0.825 |
| GraphBind | 0.933 | **0.624** | 0.293 | 0.399 | 0.392 | 0.904 |
| **GraphSite** | 0.958 | 0.517 | **0.354** | **0.420** | **0.397** | **0.917** |

Supplementary Table 5: Summary of protein-ligand binding sites datasets

| Datasets | Protein-RNA | | Antibody-antigen | |
| --- | --- | --- | --- | --- |
|  | TR545 | TE161 | TE1011 | TE259 |
| Ligand-binding residues | 18559 | 6966 | 15749 | 3755 |
| Non-binding residues | 171879 | 44349 | 189519 | 50890 |
| % of binding residues | 9.75 | 13.58 | 7.67 | 6.87 |

Supplementary Table 6: The performance of CLAPE for other ligand-binding sites prediction.

| Models | F1 | MCC | AUC | AUPR |
| --- | --- | --- | --- | --- |
| CLAPE-RB | 0.495 | 0.407 | 0.830 | 0.511 |
| CLAPE-AB | 0.567 | 0.534 | 0.920 | 0.568 |

Supplementary Table 7: Summary of protein-RNA binding sites datasets TR495 and TE117.

| Dataset | TR495 | TE117 |
| --- | --- | --- |
| DNA-binding residues | 14609 | 2031 |
| Non-binding residues | 122290 | 35314 |
| % of binding residues | 10.76 | 5.44 |

Supplementary Table 8: Comparison of CLAPE-RB with other RNA-biding sites prediction tools on TE117

| Models | Rec | Pre | F1 | MCC | AUC |
| --- | --- | --- | --- | --- | --- |
| RNABindPlus | 0.273 | 0.227 | 0.248 | 0.202 | 0.717 |
| SVMnuc | 0.231 | 0.240 | 0.235 | 0.192 | 0.729 |
| COACH-D^*^ | 0.221 | 0.252 | 0.235 | 0.195 | 0.663 |
| NucBind^*^ | 0.231 | 0.235 | 0.233 | 0.189 | 0.715 |
| aaRNA^*^ | 0.484 | 0.166 | 0.237 | 0.214 | 0.771 |
| NucleicNet^*^ | 0.371 | 0.201 | 0.261 | 0.216 | 0.788 |
| GraphBind^*a^ | 0.303 | 0.171 | 0.218 | 0.168 | 0.718 |
| GraphBind_­_^*b^ | 0.463 | **0.294** | **0.358** | **0.322** | **0.854** |
| **CLAPE-RB** | **0.467** | 0.201 | 0.281 | 0.240 | 0.800 |

^*^ indicates structure-based models ^a^ indicates results using predicted protein structures ^b^ indicates results using experimental protein structures. Results were obtained from Xia et al[^23^](#_ENREF_23).

Supplementary Table 9: Comparison of different data augmentation approaches

| Method | AUC |
| --- | --- |
| SMOTE | 0.869 |
| Random noise | 0.845 |
| Nearest neighbor | 0.864 |

Supplementary Table 10: Comparison of ProBert with ESM-v2 as the feature extractor of CLAPE-DB

| Feature extractor | Dataset | AUC | AUPR |
| --- | --- | --- | --- |
| ProtBert | TE46 | 0.871 | 0.463 |
| ESM-2 | TE46 | 0.888 | 0.517 |
| ProtBert | TE129 | 0.881 | 0.411 |
| ESM-2 | TE129 | 0.908 | 0.528 |

Supplementary Table 11: Model performance after fine-tuning ESM-2

| Feature extractor | Dataset | AUC | AUPR |
| --- | --- | --- | --- |
| ESM-2 | TE46 | 0.888 | 0.517 |
| ESM-2 (Fine-tune) | TE46 | 0.894 | 0.525 |
| ESM-2 | TE129 | 0.908 | 0.528 |
| ESM-2 (Fine-tune) | TE129 | 0.913 | 0.497 |

Supplementary Table 12: Comparison of triplet center loss and InfoNCE loss

| Contrastive loss type | Dataset | AUC | AUPR |
| --- | --- | --- | --- |
| TCL | TE46 | 0.871 | 0.463 |
| InfoNCE | TE46 | 0.867 | 0.451 |
| TCL | TE129 | 0.881 | 0.411 |
| InfoNCE | TE129 | 0.873 | 0.408 |
